# Prediction of mortality in severe dengue cases

**DOI:** 10.1186/s12879-018-3141-6

**Published:** 2018-05-21

**Authors:** Saiful Safuan Md-Sani, Julina Md-Noor, Winn-Hui Han, Syang-Pyang Gan, Nor-Salina Rani, Hui-Loo Tan, Kanimoli Rathakrishnan, Mohd Azizuddin A-Shariffuddin, Marzilawati Abd-Rahman

**Affiliations:** 1 Department of Medicine, Hospital Kuala Lumpur, Jalan Pahang, 50586 Kuala Lumpur, Malaysia; 2 Faculty of Medicine, Universiti Teknologi MARA (UiTM), Jalan Hospital, 47000 Sungai Buloh, Selangor Malaysia; 3 Clinical Research Centre, Hospital Kuala Lumpur, Jalan Pahang, 50586 Kuala Lumpur, Malaysia

**Keywords:** Severe dengue, Mortality, Predict

## Abstract

**Background:**

Increasing incidence of dengue cases in Malaysia over the last few years has been paralleled by increased deaths. Mortality prediction models will therefore be useful in clinical management. The aim of this study is to identify factors at diagnosis of severe dengue that predicts mortality and assess predictive models based on these identified factors.

**Method:**

This is a retrospective cohort study of confirmed severe dengue patients that were admitted in 2014 to Hospital Kuala Lumpur. Data on baseline characteristics, clinical parameters, and laboratory findings at diagnosis of severe dengue were collected. The outcome of interest is death among patients diagnosed with severe dengue.

**Results:**

There were 199 patients with severe dengue included in the study. Multivariate analysis found lethargy, OR 3.84 (95% CI 1.23–12.03); bleeding, OR 8.88 (95% CI 2.91–27.15); pulse rate, OR 1.04 (95% CI 1.01–1.07); serum bicarbonate, OR 0.79 (95% CI 0.70–0.89) and serum lactate OR 1.27 (95% CI 1.09–1.47), to be statistically significant predictors of death. The regression equation to our model with the highest AUROC, 83.5 (95% CI 72.4–94.6), is: Log odds of death amongst severe dengue cases = − 1.021 - 0.220(Serum bicarbonate) + 0.001(ALT) + 0.067(Age) - 0.190(Gender).

**Conclusion:**

This study showed that a large proportion of severe dengue occurred early, whilst patients were still febrile. The best prediction model to predict death at recognition of severe dengue is a model that incorporates serum bicarbonate and ALT levels.

**Electronic supplementary material:**

The online version of this article (10.1186/s12879-018-3141-6) contains supplementary material, which is available to authorized users.

## Background

Dengue infection has occurred in Malaysia for over a century [1]. Since the 1980s, outbreaks have been increasing in frequency [2]. Dengue is now accepted as endemic in Malaysia for the last 20 years. Despite massive efforts by various central government departments managing public health and many other agencies of the local government to curb it, the number of dengue cases reported continues to increase, and a rise in mortality has paralleled that increase. A national key performance indicator was established; a case fatality rate of less than 0.2% was targeted.

An epidemiological analysis in Malaysia revealed that all four DENV serotypes were found to be co-circulating during the period 2000–2012, although the predominant serotypes varied over time, both nationally and within the individual states in Malaysia [3]. An example of this variation occurred in the year 2014 when the predominant serotype had switched twice from DENV-2 to DENV-1, in February and June [4]. Prior to 2013, the case fatality rates were less than 0.2%, which was within the national target. However, from 2013 onwards, dengue incidence and case fatality rate started to increase and in 2015 had almost doubled [5].

Along with this increase in burden of disease the management of dengue, especially severe dengue, continues to baffle physicians. Despite continuous efforts to upgrade and improve clinical practice guidelines, physicians are confronted with patients who do not fall neatly into typical description of the disease, hence pose a clinical conundrum. The spectrum of dengue infection ranges from asymptomatic to mild febrile illness, dengue with warning signs and severe dengue. Severe dengue is defined as end organ involvement, severe bleeding and/or severe plasma leakage. Although only a small percentage of patients went on to develop severe dengue, with a study quoting a proportion of 5% of all dengue infections studied [6], these severe dengue carries significant mortality. Mortality in dengue is believed to be potentially preventable. Dengue is a dynamic, systemic illness and physicians have always found themselves summing up best estimates based on personal experience.

In that respect, additional knowledge would be indispensable in the management of dengue patients. Many studies have attempted to predict those that will develop severe dengue using clinical features and laboratory findings. There have also been studies that examined inflammatory markers, cytokines and genetic markers in predicting severity of dengue [7]. However, we believe that there is a need to refine this further - to predict mortality among those diagnosed to have severe dengue. We therefore aimed to identify factors at diagnosis of severe dengue that predict mortality and, built and assess predictive models based on these identified factors.

## Methods

The study was approved by the Medical Research and Ethics Committee (MREC), Ministry of Health of Malaysia (Research ID NMRR-15-2023-24,849). As the study involved data collection from case notes only, the MREC granted a waiver of informed consent. Our report is based on the STrengthening the Reporting of OBservational studies in Epidemiology (STROBE) 2015 guideline.

The study was a retrospective study of adult patients with severe dengue who were admitted into Hospital Kuala Lumpur throughout 2014. Sample recruitment was conducted from 1 January 2014 until 31 December 2014. Data were extracted from case notes of selected patients who fulfilled the study’s inclusion criteria. A data collection pro forma was used to ensure integrity of data. As the information in the case notes were information used in patients’ management, and thus would have been corrected for errors by managing clinicians, therefore we considered the information in the case notes as accurate.

Patients were selected for inclusion if, 1) they were ≥ 18 years old, 2) their presentation satisfied our criteria for suspected dengue, 3) the case fulfilled our definition of severe dengue, and 4) the presence of dengue viral infection was confirmed via NS1 antigen, high-titre level of IgG or positive IgM, from an admission serum sample. Our criteria for suspected dengue, based on the World Health Organization (WHO) 2009 criteria, were fever plus any two of: 1) aches and pain, 2) nausea and/or vomiting, 3) rash, 4) leucopenia, or 5) presence of any warning signs. The definition of severe dengue in our study was also based on WHO 2009 definition. We defined severe dengue by presence of any one of the following: 1) decompensated shock due to severe plasma leakage, 2) compensated shock due to severe plasma leakage, 3) respiratory compromise due to severe plasma leakage, 4) severe hepatitis, 5) severe bleeding that required intervention, or 6) severe organ involvement such as acute kidney injury defined by elevated serum creatinine (according to gender-specific levels), myocarditis or encephalopathy. Decompensated shock was defined by presence of systolic blood pressure (SBP) less than 90 mmHg, or mean arterial pressure (MAP) of less than 65 mmHg, or a drop in systolic blood pressure of more than 40 mmHg from patient’s known usual baseline readings. Compensated shock required signs of impaired peripheral perfusion, occurring in combination rather than singly, in presence of systolic blood pressure of ≥90 mmHg. Severe hepatitis was defined as AST level ≥ 1000 IU/L, or ALT level ≥ 1000 IU/L.

Clinical management at Hospital Kuala Lumpur followed the Malaysian Clinical Practice Guideline on Management of Dengue Infection in Adults 2010 and the WHO 2009 clinical practice guideline for dengue.

Our outcome of interest was mortality among patients diagnosed with severe dengue. Patients were grouped into those who died and those who survived. We reviewed case notes to collect data. Data collected were baseline characteristics, clinical parameters, laboratory findings and time of events. Times of events were: dates and time of fever onset, time of admission, time of diagnosis of severe dengue, time of defervescence (start of temperature persistently < 38 °C) and time of occurrence of outcome. Clinical and laboratory parameters that were closest to the time of diagnosis of severe dengue were collected. These variables formed as candidate predictors for our prediction model. We also collected data on nadir and highest levels of relevant laboratory parameters, along with their timings, for descriptive purposes. Data on types of severe dengue diagnoses that occurred in a patient were also collected.

We calculated that a sample size of 13 deaths was needed for this study for an AUROC of 70%, at confidence level of 95% and power of 80%. Analyses were performed in SAS University Edition (Copyright © 2012–2016, SAS Institute Inc., Cary, NC, USA). Continuous variables were tested for uniformity using the Kolmogorov-Smirnov test and normality with the Shapiro-Wilk test. As our data were mostly non-parametric, we used non-parametric analyses for data interrogation. Categorical variables were expressed as frequencies and percentages. Continuous variables with non-normal distribution were summarised as median and inter-quartile range (IQR).

Identification of all factors that were significantly associated with mortality was made using the Chi-square test or Fisher’s exact test for categorical variables and Mann–Whitney U test for continuous variables. We then performed multivariate analysis, with age and gender adjustments, to determine factors independently associated with mortality.

In order to build a predictive model, we used variables at the time of diagnosis of severe dengue as base for variable selection in model-building. Variable selection was performed using five-fold cross-validated Lasso regression. Selected variables were then used to build logistic regression models with cross-validation. Cross-validation and Lasso addressed overfitting that is known to occur in logistic regression. Logistic regression models built were composed of a combination of 2 of the Lasso-selected variables; and were adjusted for age and gender. All possible combinations were built. Finally, their AUROC were computed to allow comparison between models.

All tests of significance were 2-sided, and we took *p*-value < 0.05 indicating statistical significance.

## Results

### Patient characteristics

A total of 199 adult patients diagnosed as severe dengue were admitted to the Department of Medicine, Kuala Lumpur Hospital between 1 January 2014 and 31 December 2015 and all were included. All cases had confirmation of dengue infection by NS1 antigen, high-titre IgG or IgM or combination. Of this, 20 patients died.

The clinical characteristics and laboratory parameters are shown in Table 1. Patients who died were significantly older than survivors (*p* = 0.0003). However, only 3% of total were aged ≥60 years old. Two-thirds of our severe dengue patients were males. More than a third (34.2%) was obese (BMI ≥ 27.5 kg/m^2^). Co-morbidity was present in almost a third but there was no statistically significant difference between groups. The commonest co-morbidity was diabetes mellitus (12.1%). Patients who died had a statistically significant larger proportion of patients with multiple co-morbidities compared to survivors (*p* = 0.04). NS1 antigen was positive in two-thirds of patients, high-titre IgG in almost a quarter and more than half were IgM positive. There was no statistically significant difference between groups.

**Table 1:** Clinical characteristics and laboratory parameters of 199 patients hospitalised with severe dengue in 2014

	All (*N* = 199)		Died (*N* = 20)		Survived (*N* = 179)		*p*-value
	*n*	n(%) or median(IQR)	*n*	n(%) or median(IQR)	*n*	n(%) or median(IQR)	
Age, years	197	30.8 (24.7–41.3)	20	43.5 (30.3–54.8)	177	30.2 (23.9–38.9)	0.0003
BMI	117	24.9 (21.3–29.7)	6	23.6 (22.7–24.1)	111	25.3 (21.3–30.0)	0.29
Gender, Male	199	127 (63.8%)	20	13 (65%)	179	114 (63.7%)	0.91
Presence of any co-morbidity	198	62 (31.2%)	20	9 (45%)	178	53 (29.6%)	0.16
Co-morbidity							
COPD/BA		9 (4.6%)		0 (0%)		9 (5.1%)	
DM		24 (12.1%)		5 (25%)		19 (10.7%)	
Hypertension		9 (4.6%)		2 (10%)		7 (3.9%)	
IHD		2 (1%)		1 (5%)		1 (0.6%)	
Pregnancy		8 (4%)		1 (5%)		7 (3.9%)	
Others		10 (5.1%)		0 (0%)		10 (5.6%)	
Multiple co-morbidities	198	20 (10.1%)	20	5 (25%)	178	15 (8.4%)	0.04^a^
NS1	198	132 (66.7%)	20	13 (65%)	178	119 (66.9%)	0.87
High-titre IgG	198	47 (23.7%)	20	5 (25%)	178	42 (23.6%)	0.89
IgM	198	115 (58.1%)	20	14 (70%)	178	101 (56.7%)	0.25
Persistent vomiting	198	114 (57.6%)	20	14 (70%)	178	100 (56.2%)	0.24
Abdominal pain	198	89 (45%)	20	7 (35%)	178	82 (46.1%)	0.35
Lethargy	198	96 (48.5%)	20	15 (75%)	178	81 (45.5%)	0.01
Palpable liver	198	16 (8.1%)	20	2 (10%)	178	14 (7.9%)	0.67^a^
Fluid accumulation	198	44 (22.2%)	20	4 (20%)	178	40 (22.5%)	1.00^a^
Bleed	198	49 (24.8%)	20	13 (65%)	178	36 (20.2%)	< 0.0001^a^
Hct > 46	198	77 (38.9%)	20	10 (50%)	178	67 (37.6%)	0.28
Low Plt < 46	198	106 (53.5%)	20	15 (75%)	178	91 (51.1%)	0.04
Albumin < 34	198	106 (53.5%)	20	13 (65%)	178	93 (52.3%)	0.28
Number of warning signs	199		20		179		NS^b^
0		9 (4.5%)		0 (0%)		9 (5.03%)	
1		17 (8.5%)		0 (0%)		17 (9.5%)	
2		35 (17.6%)		2 (10%)		33 (18.4%)	
3		35 (17.6%)		4 (20%)		31 (17.3%)	
4		49 (24.6%)		6 (30%)		43 (24.0%)	
5		29 (14.6%)		2 (10%)		27 (15.1%)	
6		16 (8%)		3 (15%)		13 (7.3%)	
7		5 (2.5%)		0 (0%)		5 (2.8%)	
8		3 (1.5%)		2 (10%)		1 (0.6%)	
9		1 (0.5%)		1 (5%)		0 (0%)	
Presence of any warning signs	198	190 (95.5%)	20	20 (100%)	178	170 (95.0%)	1.00^a^
Severe dengue by type							
Decompensated shock		58 (29.1%)		10 (50%)		48 (26.8%)	
Compensated shock		69 (34.7%)		4 (20%)		65 (36.3%)	
Respiratory compromise		42 (21.1%)		2 (10%)		40 (22.3%)	
Severe bleeding		24 (12.1%)		10 (50%)		14 (7.8%)	
Severe hepatitis		40 (20.1%)		8 (40%)		32 (17.9%)	
AKI		35 (17.6%)		8 (40%)		27 (15.1%)	
Encephalitis		6 (3.0%)		2 (10%)		4 (2.2%)	
Other		5 (2.5%)		1 (5%)		4 (2.2%)	
Number of types of severe dengue							NS^b^
1		139 (69.8%)		12 (60%)		127 (70.9%)	
2		53 (26.6%)		4 (20%)		49 (27.4%)	
3		5 (2.5%)		3 (15%)		2 (1.1%)	
4		2 (1.0%)		1 (5%)		1 (0.6%)	
Blood products given	198	57 (28.8%)	20	18 (90%)	178	39 (21.9%)	< 0.0001
SBP, mmHg	198	103 (90–120)	20	98 (87–133)	178	103 (90–118)	0.88
DBP, mmHg	198	63 (53–74)	20	60.5 (48–84)	178	63 (54–74)	0.92
MAP, mmHg	198	77 (65–89)	20	74.3 (61–102)	178	77.3 (66–88)	0.96
PR, beats per minute	197	98 (86–110)	20	110 (102–120)	177	96 (86–109)	0.01
Shock Index, bpm/mmHg	197	1.2 (1.0–1.5)	20	1.3 (1.0–1.9)	177	1.2 (1.0–1.4)	0.25
RR, breaths/min	182	20 (18–24)	20	22 (19–30)	162	20(18–23)	0.07
WBC, ×10^3^/μL	198	4.2 (2.9–5.7)	20	5.3 (2.7–8.4)	178	4.1 (2.9–5.6)	0.15
Hb, g/dL	198	14.2(12.3–16.2)	20	13.9 (11.7–17.1)	178	14.2 (12.3–16.1)	0.97
Hct, %	198	42 (37–47.0)	20	42.5 (31.0–49.5)	178	42 (37.0–47.0)	0.91
Platelet, × 10^3^/μL	197	43 (16–97)	20	26 (8–41)	177	53 (18–105)	0.005
Serum creatinine, μmol/L	195	79 (63–101)	20	110 (67–333)	175	75 (63–97)	0.01
AST, U/L	184	165 (66–600)	19	1175 (123–1920)	165	152 (63–456)	0.01
ALT, U/L	192	104 (34–257)	20	365 (62–755)	172	95 (31–211)	0.01
Troponin T, ng/mL	17	0.02 (0.009–0.3)	6	0.4 (0.05–1.9)	11	0.009 (0.007–0.03)	0.10
Serum bicarbonate, mmol/L	192	21.1 (18.5–23.1)	20	16.2 (11.2–18.8)	172	21.5 (19.4–23.2)	< 0.0001
Serum lactate, mmol/L	149	1.6 (1.1–2.3)	19	3.2 (2.1–6.5)	130	1.5 (1.1–2.1)	< 0.0001
Nadir WBC, × 10^3^/μL	198	2.7 (1.94–3.8)	20	2.6 (2.1–4.1)	178	2.7 (1.9–3.7)	0.47
Highest Hct, %	198	45.1 (40.9–49.0)	20	46.9 (41.0–50.2)	178	45.0 (40.9–49.0)	0.38
Nadir Platelet, × 10^3^/μL	198	18 (7–44)	20	7 (2–21)	178	19 (8–45)	0.005
Highest serum creatinine, μmol/L	198	87 (69–112)	20	249 (175–378)	178	83 (68–102)	< 0.0001
Highest AST, U/L	193	262 (100–1024)	20	4763 (658–21,547)	173	244 (98–589)	< 0.0001
Highest ALT, U/L	197	147 (58–450)	20	1243 (243–3780)	177	117 (55–303)	< 0.0001
Highest Troponin T, ng/mL	22	0.01 (0.005–0.3)	5	0.7 (0.1–1.9)	17	0.01 (0.005–0.03)	0.07
Lowest Serum bicarbonate, mmol/L	195	19.5 (16.9–21.4)	20	8.5 (6.7–13.1)	175	19.9 (17.9–21.5)	< 0.0001
Highest Serum lactate, mmol/L	189	1.9 (1.3–2.8)	20	16.0 (6.7–18.5)	169	1.8 (1.2–2.3)	< 0.0001
Total fluids given, mL	193	6034 (3470–8707)	20	9808 (7184–16,692)	173	5610 (3307–8259)	0.0004

*n* count of non-missing observations; ^a^Fisher’s exact test; ^b^Post-hoc multiple comparisons tests using Scheffe’s method; *IQR* interquartile range, *BMI* body mass index, *COPD* chronic obstructive airway disease, *BA* bronchial asthma, *DM* diabetes mellitus, *IHD* ischemic heart disease, *NS1* non-structural protein 1 antigen, *SBP* systolic blood pressure, *DBP* diastolic blood pressure, *MAP* mean arterial pressure, *PR* pulse rate, *RR* respiratory rate, *AST* aspartate transaminase, *ALT* alanine transaminase, *WBC* white blood cell count, *Hct* hematocrit, *AKI* acute kidney injury, *NS* not significant

### Proportions of abnormal parameters at diagnosis of severe dengue

Systolic blood pressure < 90 mmHg was present in 22.2% of patients and there were 16.2% of patients with SBP < 90 mmHg who were still febrile (temperature > 38 °C). Mean arterial pressure < 65 mmHg was present in 22.7%, diastolic blood pressure < 50 mmHg in 18.7% and tachycardia defined by pulse rate > 100 beats/min in 47.7%. The proportion of patients diagnosed with shock who had temperature > 38 °C were 38.9%. Respiratory rate ≥ 25 breaths per minute occurred in 18.1% of patients.

Leucopenia was present only in 46% of patients and platelet count < 20 × 10^3^/μL in 27.9%. Aspartate transaminase ≥1000 U/L occurred in 21.7% of patients and alanine transaminase ≥1000 U/L in merely 4.2%. Serum bicarbonate was abnormal (< 21 mmol/L) in 48.4% of patients whilst serum lactate > 2.0 mmol/L was detected in 36.9%.

Haematocrit was elevated in 41.3% of males (Hct > 46%) and 43.1% of females (Hct > 40%). Serum creatinine was elevated in 27.2% of males (serum creatinine > 106 μmol/L) and in 11.4% of females (serum creatinine > 96 μmol/L).

### Temporal relationships

Timing of clinical events and phases of clinical course are shown in Table 2. Median admission to outcome duration was 8.75 days. Timings of admission, diagnosis of severe dengue, defervescence and outcome occurred earlier in patients who died compared to survivors; however these were not statistically significant. Our study revealed that majority of patients were still febrile (temperature were > 38 °C) when they were admitted and when they were diagnosed as severe dengue. These findings were statistically significantly different between groups. Over a third of patients presented as severe dengue upon presentation but no difference were seen between groups.

**Table 2:** Timing of clinical events in 199 patients hospitalised with severe dengue in 2014

	All (*N* = 199)		Died (*N* = 20)		Survived (*N* = 179)		*p*-value
	*n*	n (%) or median (IQR)	*n*	n (%) or median (IQR)	*n*	n (%) or median (IQR)	
^*1*^Day of admission	199	4.00 (2.81–5.00)	20	3.51 (2.58–4.38)	179	4.03 (2.93–5.00)	0.29
^*2*^Day of development of severe dengue	198	4.63 (3.56–5.67)	20	4.07 (3.22–5.81)	178	4.65 (3.66–5.67)	0.39
^*3*^Day of defervescence	194	4.96 (3.92–6.04)	18	4.08 (3.00–5.13)	176	5.02 (4.03–6.13)	0.05
^*4*^Day of outcome	199	8.75 (7.16–11.0)	20	6.81 (4.91–12.82)	179	8.80 (7.46–10.67)	0.08
Phase at admission, febrile	194	153 (78.8)	18	10 (55.6)	176	143 (81.3)	0.03^a^
Phase at severe dengue diagnosis, febrile	193	114 (59.1)	18	6 (33.3)	175	108 (61.7)	0.02
Severe dengue upon presentation	198	73 (36.9)	20	8 (40.0)	178	65 (36.5)	0.76
^*5*^Day of nadir WBC	199	4.63 (3.75–5.71)	20	3.74 (2.93–5.34)	179	4.71 (3.88–5.73)	0.07
^*6*^Day of nadir platelet	199	5.50 (4.64–6.50)	20	4.79 (3.40–6.96)	179	5.54 (4.79–6.50)	0.11
^*7*^Day of lowest serum bicarbonate	197	5.00 (3.92–6.04)	20	5.36 (3.56–8.94)	177	4.92 (3.96–5.92)	0.15
^*8*^Day of highest serum lactate	190	5.38 (4.13–6.60)	20	5.44 (3.93–9.70)	170	5.35 (4.13–6.50)	0.32
^*9*^Day of highest serum ALT	198	5.56 (4.42–6.75)	20	5.41 (4.05–7.02)	178	5.58 (4.42–6.75)	0.88
^*10*^Day of highest serum AST	194	5.35 (4.29–6.54)	20	5.26 (4.18–7.39)	174	5.35 (4.38–6.50)	0.81
^*11*^Day of highest serum creatinine	199	4.67 (3.67–5.79)	20	4.81 (4.00–8.38)	179	4.67 (3.58–5.71)	0.10

*n* count of non-missing observations; ^a^Fisher’s exact test; *IQR* interquartile range, *WBC* white blood cell count, *AST* aspartate transaminase, *ALT* alanine transaminase; *1–11* These are days from onset of fever

We also found that nadir platelet occurred almost a day later after nadir WBC in both groups. However, these timings were not statistically significantly different between groups. Similarly, lowest serum bicarbonate occurred before serum lactate peaked however no statistical difference between groups in terms of timings were found.

### Factors associated with death: Univariate analysis

Univariate analyses found 22 clinical and laboratory parameters which were statistically significantly different between patients who died compared to those who survived (Tables 1 and 2). Factors at diagnosis of severe dengue which were associated with mortality were: older age, presence of multiple co-morbidities, presence of lethargy, bleeding, tachycardia, lower platelet count, elevated serum creatinine, elevated AST, elevated ALT, low serum bicarbonate and elevated serum lactate.

Our univariate analyses also showed that mortality was also associated with lower nadir platelet level. In the group that died, highest median serum creatinine was 3 times higher than survivors, highest median AST was almost 20 times higher, highest median ALT was more than 10 times higher, highest median lactate was more than 8 times higher and lowest median serum bicarbonate was more than 2-fold lower. These had statistically significant difference between groups. Non-survivors were found to have received statistically significantly higher volume of fluid (1.7 times more) and a larger proportion (4.1 times) who received blood products transfusion.

### Factors associated with death: Multivariate analysis and predictive model

Multivariate analysis, with adjustment for age and gender, revealed lethargy, bleeding, pulse rate, serum bicarbonate and serum lactate, to be statistically significant as independent predictors of death among severe dengue cases (Table 3).

**Table 3:** Multivariate analysis of potential factors that predict mortality at the time of diagnosis of severe dengue

Parameters	Adjusted OR	95% CI	*p*
Lethargy	3.84	(1.23–12.03)	0.0208
Bleed	8.88	(2.91–27.15)	0.0001
Multiple co-morbidities	1.28	(0.32–5.15)	0.7258
Pulse rate	1.04	(1.01–1.07)	0.0058
Serum bicarbonate	0.79	(0.70–0.89)	< 0.0001
Serum lactate	1.27	(1.09–1.47)	0.0019
Plt	0.981	(0.97–1.00)	0.0231
ALT	1.001	(1.00–1.002)	0.0046
AST	1.001	(1.00–1.001)	0.0152
Serum creatinine	1.008	(1.00–1.01)	0.0044

*OR* odds ratio, *CI* confidence interval

**Fig. 1:**
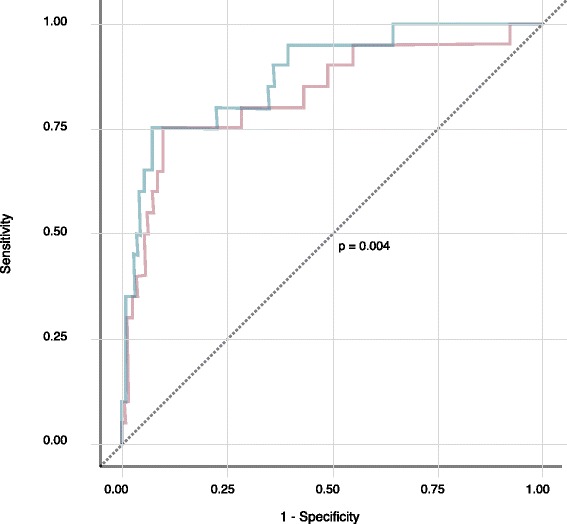
Cross-validated and uncross-validated (basic) ROC curves of the serum bicarbonate - ALT model

Lasso selected age, pulse rate, bleeding, serum bicarbonate, serum lactate, serum creatinine, AST and ALT out of 29 candidate variables. Pulse rate, bleeding, serum bicarbonate, serum lactate, serum creatinine, AST and ALT were then used to build models composed of a combination of a pair of these variables, with age and gender adjustments. Twenty-one models were built (Additional file 1: Table S1). We found that serum bicarbonate-based models outperformed lactate-based models (Table 4). The best AUROC was obtained with serum bicarbonate - ALT model. The cross-validated serum bicarbonate - ALT model was found to be significantly different from the uncrossed model (Fig. 1).

**Table 4:** Area under curve of receiver operating curve (AUROC) of models for predicting mortality among cases of severe dengue

Model	AUROC (95% CI)
Serum bicarbonate - ALT	83.5 (72.4–94.6)
Serum bicarbonate - Pulse rate	83.4 (73.5–93.4)
Serum bicarbonate - Serum creatinine	83.1 (72.0–94.3)
Serum bicarbonate - Bleed	82.9 (70.5–95.2)
Serum lactate - ALT	80.8 (67.2–94.4)
Serum lactate - Pulse rate	78.7 (65.7–91.8)
Serum lactate - Serum creatinine	77.7 (63.9–91.5)
Serum lactate - Bleed	81.1 (68.2–94.0)

*AUROC* area under curve; *CI* confidence interval

## Discussion

Our study made two notable findings: the first being description of aspects of severity of a severe dengue cohort; and the second, the rigorous building of a predictive model, which was selected by assessment and comparison of many models, that predicts death early at the recognition of severe dengue.

Our cohort bears some characteristics of Malaysia’s population. The National Health Morbidity Survey 2015 determined that 30.6% of the population were obese and 17.5% were diabetics, figures similar to this study [8].

The fatality rate of this study was 10.1%. Previous studies similar to ours had rates of 3.8% [9] and 18.6% [10] in Brazil. Amâncio et al. [10] non-survivors had median (IQR) AST 151 (43–474) U/L and mean (SD) platelet count 83.3 × 10^3^/μL (76.2 × 10^3^/μL); the other study [9] however, had no similar parameters for comparison. These levels were worse in our study. Other studies, which used ICU admission as the defining criterion of their cohorts, had case fatality rates of 23.1% [11] in Taiwan whilst in India rates were 11.1% [12] and 6.1% [13]. Chen et al. [11] had non-survivors with mean (SD) AST 3444.4 (4191.9) U/L and mean (SD) nadir platelet 3.5 × 10^3^/μL (4.3 × 10^3^/μL). In contrast, the non-survivors of our study had even higher peak AST level, suggesting a more severe cohort in ours.

Our study showed that about a third of patients presented as severe dengue upon admission to hospital whilst the remaining developed severe dengue after admission. Duration of fever onset to severe dengue diagnosis were shorter in those who presented as severe dengue as compared to those who developed severe dengue after admission, median(IQR) 4.38 [2] days vs median(IQR) 5.02(1.78) days, *p* = 0.003, respectively. Interestingly, there were no difference in mortality and in the duration of fever onset to admission between those who presented as severe dengue as compared to those who developed severe dengue after admission, *p* = 0.95 vs *p* = 0.29, respectively.

Guidelines in dengue have repeatedly highlighted that the timing of deterioration is around the time of defervescence and within the critical phase that ensues. It has also been noted that organ impairment follows the same timing. Intriguingly, we found that almost 60% of patients were still febrile at diagnosis of severe dengue; and the proportion of patients who were still febrile at diagnosis of severe dengue was statistically significantly more in those who survived as compared to those who died (Table 2). In fact, those who were febrile at diagnosis of severe dengue were less likely to die as compared to those who had defervesced, OR 0.29 (95% CI: 0.09–0.92, *p* = 0.03). The importance of timing of development of severe dengue in the course of illness needs to be further explored.

Our findings that nadir platelet occurred about a day after nadir WBC is consistent with a previous study [14]. However, another intriguing finding of our study is that the timing of peak serum creatinine coincided with nadir WBC in those who survived whereas in those who died it coincided with the later nadir platelet.

Based on our cohort, independent predictors of death at the time when the diagnosis of severe dengue was made were: lethargy, bleeding, pulse rate, serum bicarbonate and serum lactate. There have been only 2 studies so far, that examined factors associated with death among severe dengue patients, which used WHO 2009 classification. A study utilising notification database from Brazil [9] of mixed age groups showed age > 55 years (OR 4.98), gastrointestinal bleeding (OR 10.26), haematuria (OR 5.07), and thrombocytopenia (OR 2.55) were factors associated with death. However, exact timings of these parameters were not included. A second study [10] was of severe dengue patients admitted to ICU in Brazil. In this study of 97 patients admitted to ICU, parameters taken within 24 h of ICU admission that were found to be associated with death were: comorbidity of chronic renal disease (OR 15.6), presence of persistent vomiting (OR 4.25), lethargy (OR 3.23), dyspnoea (OR 3.27), elevated WBC, higher serum creatinine and lower serum albumin.

We found that the best prediction model to predict death at the time when the diagnosis of severe dengue was made is a model that incorporated serum bicarbonate and ALT levels taken at that time. This is rather fortuitous for a few reasons. Firstly, both serum bicarbonate and ALT are objective measures as opposed to subjective warning signs such as lethargy and bleeding which have inherent variability in establishing their presence and severity. Secondly, both are currently accessible laboratory tests.

A recent study on serum lactate in dengue [15] found that this biomarker is a good predictor of severe dengue (AUROC for peripheral venous lactate at admission was 0.84 [95% CI: 0.72–0.97]). In perspective, our study revealed that in predicting death, lactate-incorporated models had lower AUROCs as compared to those of bicarbonate-based models (Table 4). Additionally, we showed that nadir serum bicarbonate occurred earlier than highest serum lactate level (Table 2) in patients with severe dengue. In fact, at diagnosis of severe dengue, more patients had abnormal serum bicarbonate than abnormal serum lactate (46.7% vs 27.6%, respectively). These are important aspects to consider in clinical practice as earlier management will be more advantageous. Therefore, whilst lactate could be an additional alternative criterion to establish the diagnosis of severe dengue, our study suggests that lactate alone is not sufficient in prognostication of patients with severe dengue.

Unexpectedly, serum creatinine was not found to be an independent predictor of death by multivariate analysis. However, its incorporation into our models led to good AUROC performances.

Under the assumption that the time of recognition of severe dengue is approximately the actual time of development of severe dengue, we believe that prognosticating mortality in patients with severe dengue at the time of its recognition provides a sensible approach. We postulate that at this time, underlying pathophysiological processes which determine outcome would most likely have reach significance. Prognosticating prematurely before this moment may have little specificity, as the ultimate outcome determining processes may yet to occur. Prognosticating too late however is obviously futile. With our model management decisions may be better informed in terms of resource allocation, especially in conditions of high volume care. It has to be noted however that the underlying outcome determining pathophysiology has yet to be clearly elucidated. Further investigation into the kinetics of biochemistry with respect to timing of events in dengue, in particular the time of development of severe dengue, is needed and may perhaps fill this gap.

The main limitation of our study was the retrospective design. However, data accuracy was reasonable as management of patients followed standard management guidelines for dengue which have clear specifications of timing of blood investigations. Though only a single centre study, we believe the sample size was adequate as illustrated by the results of our findings. Finally, though we have rigorously built a predictive model and took steps to address overfitting, the actual performance of any model will vary according to the population it is applied on. As we have mentioned, our cohort is different from similar studies, hence our model will require population specific external validation and assessment.

## Conclusions

In conclusion, the degree of severity observed in this study was more serious than those reported in similar studies. We showed that a large proportion of severe dengue occurred early, whilst patients were still febrile. Finally, the regression equation to our model is: Log odds of death among severe dengue cases = − 1.021 - 0.220(Serum bicarbonate) + 0.001(ALT) + 0.067(Age) - 0.190(Gender).

## Additional file

Additional file 1: **Table S1.** AUROC of all logistic regression model combinations. The table contains areas under curve of receiver operating curves (AUROC) of all logistic regression models built using pairwise combinations of LASSO-selected variables. All possible pairwise combinations were made. Each model was adjusted for age and gender. Models were listed from highest to lowest AUROC. (DOCX 9 kb)

## Abbreviations

ALT: Alanine transaminase
AST: Aspartate transaminases
AUROC: Area under curve of receiver operating curve
CI: Confidence interval
DENV: Dengue virus
ICU: Intensive care unit
IgG: Immunoglobulin G
IgM: Immunoglobulin M
IQR: Interquartile range
MAP: Mean arterial blood pressure
MREC: Medical Research and Ethics Committee
NMRR: National Medical Research Register
NS1: Non-structural protein 1
OR: Odds ratio
SBP: Systolic blood pressure
SD: Standard deviation
STROBE: STrengthening the Reporting of OBservational studies in Epidemiology
WBC: White blood count
WHO: World Health Organization
