# Supplementary material for: Prediction of mortality in severe dengue cases
Source: BMC Infect Dis. 2018 May 21;18:232. doi: 10.1186/s12879-018-3141-6 (PMC5963083; doi:10.1186/s12879-018-3141-6)
Supplement: Supplementary file 1 — Table S1. AUROC of all logistic regression model combinations. The table contains areas under curve of receiver operating curves (AUROC) of all logistic regression models built using pairwise combinations of LASSO-selected variables. All possible pairwise combinations were made. Each model was adjusted for age and gender. Models were listed from highest to lowest AUROC. (DOCX 9 kb) [file 12879_2018_3141_MOESM1_ESM.docx]

Table S1. AUROC of all logistic regression model and combinations.

| **Logistic models (**including age & gender as covariates for each model**)** | **Unvalidated AUROC** | **Cross-validated AUROC** |
| --- | --- | --- |
| **HCO_3_, ALT** | 0.881 | 0.835 |
| **HCO_3_, Pulse rate** | 0.887 | 0.834 |
| **HCO_3_, Serum creatinine** | 0.866 | 0.831 |
| **Bleed, HCO_3_** | 0.880 | 0.829 |
| **HCO_3_, AST** | 0.865 | 0.828 |
| **Bleed, Pulse rate** | 0.867 | 0.821 |
| **Lethargy, HCO_3_** | 0.873 | 0.819 |
| **Bleed, Lactate** | 0.859 | 0.811 |
| **Lactate, ALT** | 0.850 | 0.808 |
| **Bleed, ALT** | 0.857 | 0.807 |
| **HCO_3_, Lactate** | 0.863 | 0.801 |
| **Lethargy, Lactate** | 0.842 | 0.800 |
| **Bleed, Serum creatinine** | 0.849 | 0.799 |
| **Lactate, Pulse rate** | 0.841 | 0.787 |
| **Lethargy, Serum creatinine** | 0.834 | 0.783 |
| **Lethargy, ALT** | 0.831 | 0.782 |
| **Bleed, AST** | 0.823 | 0.780 |
| **Lethargy, AST** | 0.820 | 0.778 |
| **Lactate, AST** | 0.836 | 0.777 |
| **Lactate, Serum creatinine** | 0.831 | 0.777 |
| **ALT, Pulse rate** | 0.8247 | 0.7756 |
| **Lethargy, Pulse rate** | 0.815 | 0.764 |
| **Serum creatinine, Pulse rate** | 0.812 | 0.762 |
| **AST, Pulse rate** | 0.8103 | 0.7590 |
| **Serum creatinine, ALT** | 0.804 | 0.750 |
| **ALT, AST** | 0.7914 | 0.7154 |
| **Serum creatinine, AST** | 0.774 | 0.704 |
| *AUROC* area under curve of receiver operating curve | | |
